# Optimizing Commercial-Scale Storage for Chinese Cabbage (*Brassica rapa* L. ssp. *Pekinensis*): Integrating Morphological Classification, Respiratory Heat Effects, and Computational Fluid Dynamics for Enhanced Cooling Efficiency

**DOI:** 10.3390/foods14050879

**Published:** 2025-03-04

**Authors:** Sung Gi Min, Timilehin Martins Oyinloye, Young Bae Chung, Won Byong Yoon

**Affiliations:** 1 Practical Technology Research Group, World Institute of Kimchi, Gwangju 61755, Republic of Korea; skmin@wikim.re.kr (S.G.M.); ybchung@wikim.re.kr (Y.B.C.); 2 Department of Food Science and Biotechnology, College of Agriculture and Life Sciences, Kangwon National University, Chuncheon 24341, Republic of Korea; oyinloyetm@kangwon.ac.kr; 3 Elder-Friendly Food Research Center, Agriculture and Life Science Research Institute, Kangwon National University, Chuncheon 24341, Republic of Korea

**Keywords:** Chinese cabbage classification, computational fluid dynamics (CFD), heat transfer, K-means clustering, storage optimization

## Abstract

This study optimized Chinese cabbage (*Brassica rapa* L. ssp. *pekinensis*) storage design by integrating K-means clustering, heat transfer analysis, and respiratory heat effects. A morphological assessment identified three clusters: class 1 (73.32 ± 3.34 cm length, 46.73 ± 2.24 cm width, 1503.20 ± 118.39 g weight), class 2 (82.67 ± 1.17 cm, 51.89 ± 2.37 cm, 2132.48 ± 127.16 g), and class 3 (89.17 ± 2.45 cm, 58.67 ± 2.77 cm, 2826.37 ± 121.25 g), with a silhouette coefficient of 0.87 confirming robust clustering. The CO_2_, relative humidity, and airflow analysis revealed hotspots and imbalances. Heat transfer modeling, incorporating respiratory heat, closely matched experimental data (RMSE < 0.54 °C), while excluding it caused deviations in storage. The validated model informed a modified geometry for scale-up CFD modeling, reducing the convergence time by 38% and the RAM usage by 30%. Three commercial storage designs were evaluated: fully filled, batch filled (50:50), and repositioned air conditioning with batch filling. The latter achieved a faster equilibrium (4.1 °C in 17 h 15 min vs. 21 h 30 min for fully packed) and improved airflow, reducing the hot zones. This study highlights the importance of integrating cabbage morphology, environmental factors, and respiratory heat into storage design to enhance cooling efficiency and product quality.

## 1. Introduction

Chinese cabbage (CC) (*Brassica rapa* L. ssp. *pekinensis*) is a highly perishable vegetable consumed worldwide due to its nutritional and economic significance [1,2]. However, its perishability poses challenges for postharvest storage, necessitating strategies to maintain its quality, extend its shelf life, and minimize losses. CC’s physiological characteristics, such as respiration and heat production, significantly impact the storage conditions. Poor management can cause excessive drying, shrinkage, and chilling injuries [3,4,5,6]. Respiratory heat contributes to localized temperature variations, accelerating spoilage and increasing microbial susceptibility [5,7]. Addressing these challenges requires innovative storage optimization approaches balancing temperature control and energy efficiency through heat and mass transfer analysis.

Computational fluid dynamics (CFD) is a powerful tool for optimizing the cold storage system by simulating the airflow and temperature distribution. Alexander et al. [8] modeled the airflow in apple storage, demonstrating CFD’s role in maintaining uniform conditions. Getahun [9] applied CFD to stacked orange pallets, showing its effectiveness in predicting temperature gradients and improving the cooling efficiency. These studies highlight CFD’s potential for enhancing postharvest storage, especially in commercial applications where experimental investigations may be costly or impractical.

Developing a scalable model for storage is crucial for industrial applications. Small-scale experimental validation refines CFD models before commercial deployment, enabling accurate heat and mass transfer predictions while reducing operational costs. Scaling up introduces complexities such as increased heat production, airflow resistance, and spatial temperature variations. Additionally, commercial-scale storage often involves batch filling, where produce is stored in stages rather than all at once, affecting airflow patterns and cooling efficiency. CFD simulations can assess the impact of batch loading and optimize storage arrangements to minimize temperature gradients and improve the overall storage performance [8].

However, modeling commercial-scale storage in CFD can be computationally demanding due to the high resolution needed for airflow and heat transfer accuracy. To mitigate this challenge, geometric simplifications are often used to reduce the computational costs while maintaining predictive accuracy [6,10]. In addition, porous media modeling is another effective approach, which represents bulk agricultural produce as a porous domain with defined flow resistance properties [10,11,12]. This method has been successfully applied to packed beds of potatoes and carrots, predicting the cooling efficiency and temperature uniformity [10,13]. Incorporating porosity models in CFD enables the efficient simulation of commercial-scale storage, optimizing airflow distribution and heat dissipation in CC storage [14].

The accuracy of CFD simulations in commercial-scale storage design depends on the precision of small-scale models and their ability to realistically represent stored produce. One often-overlooked factor in CC storage is the variability in cabbage size, which significantly impacts the cooling efficiency and airflow patterns. Unlike more uniformly shaped fruits, such as apples or oranges, CC exhibits wide morphological variations in length, circumference, and weight. Ignoring these differences may cause cooling inefficiencies and airflow resistance, leading to suboptimal storage conditions. To address this, K-means clustering was introduced to classify CC based on morphological properties. K-means clustering is widely used in agriculture and food systems for categorization and decision-making. For instance, Worasawate et al. [15] used it to classify mangoes based on the size and ripeness, optimizing the packaging and storage. Similarly, strawberries were graded based on their shape using clustering techniques [16]. Applying K-means clustering to CC enables a classification into distinct clusters based on length, thickness, and weight, ensuring that the size variability is considered in storage design. This approach improves airflow and temperature distribution predictions, enhancing the storage efficiency.

Therefore, the objectives of this study were as follows: (1) to classify CC based on their morphological properties, i.e., length circumference, width circumference, and weight, using the K-means clustering algorithm, thereby identifying distinct clusters for optimized storage design; (2) to develop a CFD-based simulation model to analyze the airflow, temperature distribution, and the effects of respiratory heat within cabbage storage facilities; (3) to incorporate the heat of respiration into the heat transfer model and validate the simulation results against experimental data to enhance the accuracy of temperature regulation predictions; and (4) to investigate the effects of different storage filling methods for CC on the temperature gradients, equilibrium temperature, and airflow patterns.

## 2. Materials and Methods

### 2.1. Sample Preparation

**Figure 1:**
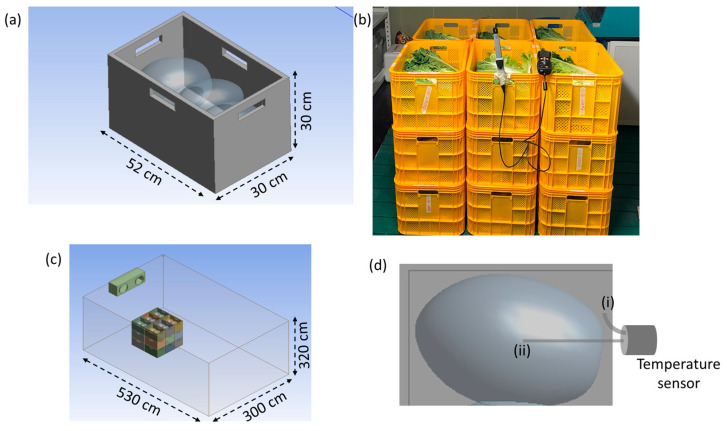
Experimental setup for the storage of Chinese cabbages (CCs): (**a**) individual storage basket dimensions; (**b**) arrangement of baskets in a 3 × 3 grid; (**c**) storage facility dimensions; and (**d**) placement of wireless thermocouples: (**i**) sensors embedded in CC to monitor internal temperature, and (**ii**) sensors positioned to record environmental temperature within the storage facility.

Freshly harvested CC were utilized in this study. A total of 162 CCs (i.e., 54 × 3 replicates) were classified based on size and weight using the K-means clustering algorithm. This classification ensured that 18 baskets each hold three CCs, containing a mix of different CC groups. By distributing CC this way, the heat generated during storage was balanced across all baskets, preventing uneven temperature effects. The baskets (52 cm × 30 cm × 30 cm, Figure 1a) were arranged in a 3 × 3 grid inside a small-scale storage facility (530 cm × 300 cm × 320 cm, Figure 1b,c). A ceiling-type unit cooler, operating with a glycol solution (37.9 kW cooling capacity and 1105 m^3^/h airflow), ensured uniform temperature distribution. Two axial fans (30 cm diameter and rotational speed of 1300 rpm) assisted in maintaining airflow.

CC circumference length, width, and weight were measured using a flexible tape and an electronic scale (±0.1 g precision). To monitor the storage environment, wireless thermocouples (Tracksense Pro, Ellab, Kongens Lyngby, Denmark) recorded CC and ambient temperatures (Figure 1(di,dii)). Testo probes (Testo 175-T2, Testo 417, and Testo 535, Testo SE & Co. KGaA, Lenzkirch, Germany) measured air temperature, humidity, airflow, and CO_2_ levels, ensuring comprehensive monitoring. The collected data on temperature, airflow, and CO_2_ concentration were used to optimize the storage environment, minimizing heat variation and improving overall storage conditions.

### 2.2. Measurement of CC Respiration Rate

**Figure 2:**
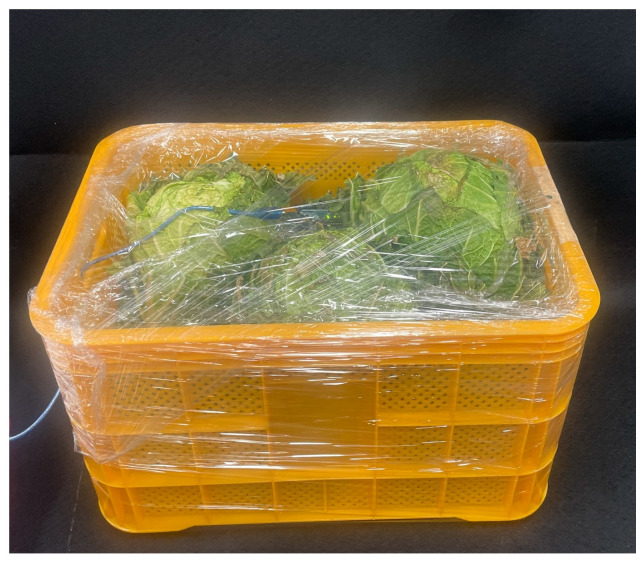
Experimental setup for measuring the respiration rate of CC.

The respiration rate of CC was measured by analyzing carbon dioxide (CO_2_) production and associated heat generation, which is critical for modeling heat and mass transfer in cold storage. To isolate the effects of respiration, three CC samples from each size class were placed in a basket and tightly wrapped with plastic film to create a sealed environment (Figure 2). This setup prevented external airflow and CO_2_ dissipation, allowing direct measurement of CO_2_ and heat generated from the CC [17,18]. Unlike open storage conditions, the sealed system ensured accurate respiration data by eliminating external interference, and it accounted for the amount of CO_2_ and heat generated as a function of the internal basket temperature [19,20].

Inside the wrapped basket, sensors for CO_2_ concentration, humidity, and temperature were installed. These sensors continuously recorded real-time data, which were used to estimate the respiration rate and its thermal effect on storage conditions. The heat released during respiration was calculated based on the CO_2_ concentration increase inside the sealed basket. The molar respiration rate of CC ($R_{{CO}_{2}}$, mol CO_2_/kg·h) was first determined using the ideal gas law [19,21]:

(1) $$R_{{CO}_{2}}=\frac{V_{chamber}\cdot ∆C_{{CO}_{2}}}{M_{cc}\cdot R\cdot T\cdot ∆t},$$

where $V_{chamber}$ is the volume of the sealed basket (m^3^), $∆C_{{CO}_{2}}$ is the change in CO_2_ concentration (ppm) over time, M_cc_ is the mass of CC in the basket (kg), R is the universal gas constant (8.314 J/mol·K), T is the absolute temperature (K), and Δt is the measurement time interval (h).

Once $R_{{CO}_{2}}$ was determined, the heat generated due to respiration (Q_T_, W/kg) was calculated using the empirical respiration heat equation from Ashrae [22]:

(2) $$Q_{T}=\frac{10.7\cdot f}{3600}{\left(9T+32\right)}^{g},$$

where T is the temperature of CC in °C, and f and g are specific respiration coefficients for CC (with f = 6.0803 × 10^−4^ and g = 2.6183). This equation links the heat produced to the temperature of the cabbage, providing an estimate of heat generation per kilogram over time [22].

The computed respiration heat (Q_T_) was used as a volumetric heat source in CFD simulations to model the heat distribution in each CC basket. The heat source term ($\dot{q}$, W/m^3^) was applied in the energy equation of the CFD model as [23,24]

(3) $$\dot{q}=\frac{Q_{T}\cdot \rho_{CC}}{V_{basket}},$$

where $\rho_{CC}$ is the bulk density of CC (Kg/m^3^) and V_basket_ is the volume of a single basket (m^3^). By incorporating $\dot{q}$ into the CFD simulation, the effect of respiration heat on airflow patterns and temperature distribution within the storage facility could be analyzed, ensuring optimal storage conditions and uniform cooling efficiency.

### 2.3. K-Means Clustering

The K-means clustering algorithm groups data into K clusters by minimizing the variance within each cluster. For a dataset with n objects (x_1_, x_2_, ⋯, x_n_), the algorithm assigns these objects to K sets (S_1_, S_2_, ⋯, S_n_), minimizing the objective function shown in Equation (4), where μ_i_ represents the centroid of cluster S_i_ [25]:

(4) $$\sum_{i=1}^{k}\sum_{x\in S_{i}}{\left|\left|x-\mu_{i}\right|\right|}^{2},$$

The optimal number of clusters (K) was determined using the elbow method, which identifies the point where additional increases in K result in negligible reductions in the sum of squared errors (SSE). To evaluate the clustering performance, silhouette index was applied. Silhouette analysis validated the clustering results, ensuring reliable grouping for cabbage classification. Based on the analysis of morphological properties (i.e., length circumference, width circumference, and weight, the optimal number of clusters was identified as K = 3. These clusters were categorized as class 1, class 2, and class 3, and they are discussed extensively in subsequent section.

### 2.4. Numerical Model

#### 2.4.1. CFD Simulation Setup

To simulate the heat transfer in CC, the cabbage’s shape was modeled as a near-spherical object, with each class represented by its average geometric dimensions. The density and thermal properties, including thermal conductivity, and specific heat, were measured experimentally. Thermal conductivity and specific heat were determined using a thermal analyzer (KD2 Pro, Decagon Devices, Pullman, WA, USA) at the storage temperature (Table 1). The density was measured using the immersion method, where the whole CC was submerged in distilled water until no air bubbles were observed, and the displaced water volume was used to calculate density [6].

The heat of respiration, calculated from CO_2_ production and temperature changes within the CC, was incorporated as a volumetric heat source within the CFD model (Section 2.2). This allowed for accurate representation of metabolic heat generation during storage. The respiration rate was integrated into the simulation as a source term in the energy equation.

#### 2.4.2. Porous Media Model

To account for the internal structure of CC and reduce computational cost, a porous media model was employed. The CC consists of an interconnected cellular matrix with voids that allow air or water vapor to flow through. This structure affects both heat transfer and airflow resistance, which must be incorporated into the model.

In the porous media approach, the CC matrix is treated as a continuous medium with effective thermal properties. The porosity (ϕ) of CC was determined experimentally using vacuum impregnation, following the method described by Zhao and Xie, [26]. The pore volume (V_p_) was calculated as

(5) $$V_{p}=\frac{{W_{saturated}-W}_{i}}{\rho_{w}},$$

where W_saturated_ is the weight after vacuum impregnation (kg), W_i_ is the initial weight (kg), and $\rho_{w}$ is the density of water. The porosity (ϕ) was then determined as

(6) $$\phi =\frac{V_{p}}{V_{i}},$$

where V_i_ is the initial volume of CC [26]. The calculated porosity was found to be 0.31.

Since the fluid in the storage environment is water vapor generated during respiration, the fluid density in the porous domain was modeled as the density of water vapor, and the dynamic viscosity is also that of water vapor. Airflow through the porous structure of CC is governed by the Darcy–Forchheimer equation [27,28]:

(7) $$\frac{\mu }{K}u+\frac{\rho_{wv}C_{F}}{\sqrt{K}}\left|u\right|u=-\nabla P,$$

where u is the velocity vector (m/s), K is permeability (m^2^), C_F_ is the Forchheimer coefficient, P is pressure (Pa), and $\rho_{wv}$ and μ are the density and viscosity of water vapor, respectively.

#### 2.4.3. Transient Heat Conduction Model

The heat transfer within the CC was modeled using the volume-averaged energy equation for porous media, which accounts for both the solid CC structure and the air within its pores. The governing equation for transient heat conduction in the porous medium is given as

(8) $$(1-\phi )\rho_{s}C_{P}^{s}+\frac{\partial T_{s}}{\partial t}+\phi \rho_{f}C_{P}^{f}\frac{\partial T_{f}}{\partial t}=\nabla \cdot (k_{eff}\nabla )+\dot{q},$$

where ϕ is porosity, $\rho_{s}$ and $\rho_{f}$ are the solid and fluid densities (kg/m^3^), $C_{P}^{s}$ and $C_{P}^{f}$ are the specific heats (J/kg·K), $T_{s}$ and $T_{f}$ are the temperatures of the solid and water vapor phases, respectively, t is time (s), $k_{eff}$ is the effective thermal conductivity (W/m·K), and $\dot{q}$ represents the heat generation rate from respiration (W/m^3^). Expanding Equation (8) in spherical coordinates, the equation becomes

(9) $$\left(1-\phi \right)\rho_{s}C_{P}^{s}\frac{\partial T_{s}}{\partial t}+\phi \rho_{f}C_{P}^{f}\frac{\partial T_{f}}{\partial t}=\frac{1}{r^{2}}\frac{\partial }{\partial r}\left(r^{2}k_{eff}\frac{\partial T}{\partial r}\right)+\frac{1}{r^{2}sin\;\theta }\frac{\partial }{\partial \theta }\left(sin\partial \theta k_{eff}\frac{\partial T}{\partial \theta }\right)+\frac{1}{r^{2}{sin}^{2}\theta }\frac{\partial^{2}T}{\partial \varphi^{2}}+\dot{q}$$

where r, θ, and φ are the radial, polar, and azimuthal coordinates in spherical coordinates (m, radians).

#### 2.4.4. Boundary Conditions

To solve the heat transfer equation for the spherical object, the following initial and boundary conditions were applied:

1. Initial condition:

(10) $$T(r,\theta ,\phi ,0)=T_{0},$$

where T_0_ is the initial temperature of CC.

2. The boundary conditions included the following:
  - Symmetry at the center (r = 0): Due to spherical symmetry, the temperature gradient at the center is zero:
    (11) $$\frac{\partial T}{\partial r}=0\;at\;r=0,$$
  - The convective heat transfer at the CC surface (r = R), modeled as a heat exchange between the cabbage and the surrounding environment (e.g., refrigeration or air):

(12) $$-k_{eff}\frac{\partial T}{\partial r}=h\left(T_{s}-T_{\infty }\right)\;at\;r=R,$$

where $k_{eff}$ is the thermal conductivity (W/m·K), h is the heat transfer coefficient (W/m^2^·K), $T_{s}$ is the surface temperature of the cabbage, and $T_{\infty }$ is the temperature of the surrounding medium (°C). This formulation ensures that heat transfer in the CC is accurately modeled by considering both its porous structure and spherical shape.

The simulation included meshing, with refinement at the CC surface for accurate heat exchange representation. Structured and unstructured meshes were used, with tetrahedral elements applied in the interior for computational efficiency. Boundary conditions were applied, and heat conduction and generation equations were solved iteratively using ANSYS (2024 version; R2, Ansys, Inc., Canonsburg, PA, USA) on a desktop with an Intel Core i9-13900K CPU (3.0 GHz) and an NVIDIA GeForce RTX 3070 GPU (24 GB GDDR6X VRAM, 10,496 CUDA cores). The model was validated by comparing simulated and experimental CC temperature data.

#### 2.4.5. Model Geometry Simplification for Commercial-Scale Storage Facility

**Figure 3:**
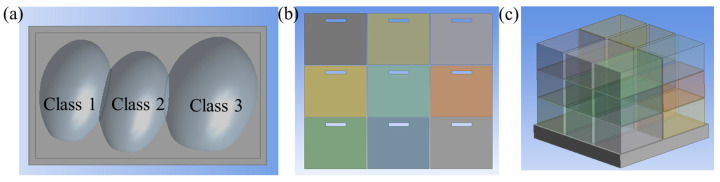
Simplification of CC geometry for commercial-scale storage modeling: (**a**) small-scale model with individual CC representations in each basket, (**b**) arranged CC baskets, and (**c**) block-based representation of baskets for commercial-scale simulation.

To facilitate computational efficiency in scaling up the model for a large storage room, a systematic simplification approach was adopted. Initially, a small-scale model was developed using the average dimensions of CC (i.e., circumference length and width) within each classification group, with each class represented in every basket (Figure 3a). To further reduce computational complexity while preserving key physical characteristics, the three CCs in each basket were unified into a single geometry model (Figure 3a). This approach effectively reduced the number of elements in the computational domain, optimizing mesh density and simulation runtime while maintaining accurate representation of heat and mass transfer properties.

The developed model, representing the average shape and size of each CC in the arranged basket, was validated against experimental results to ensure its accuracy in replicating thermal behaviors (Figure 3b). Once the model criteria were confirmed in the small-scale setup, the geometry was further simplified into a block-shaped representation of individual baskets (Figure 3c). This block-based model, upon validation at the small-scale level, was subsequently used in commercial-scale simulations to analyze heat transfer phenomena in extensive storage conditions.

### 2.5. Optimization of Commercial-Scale CC Storage Room Design

**Figure 4:**
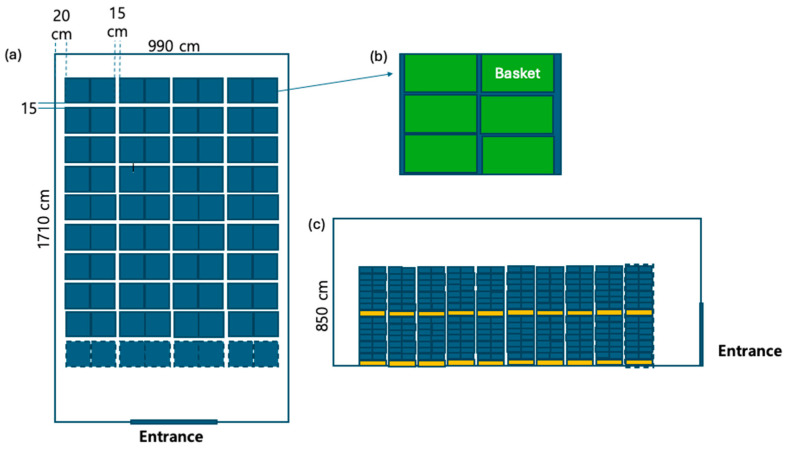
Standard storage design for cold storage of CC, showing the overall layout and configuration. (**a**) Storage dimensions, (**b**) pallet arrangement, and (**c**) cabbage box arrangement.

The optimization of the commercial cold storage facility for CC was conducted using CFD simulations informed by experimental data. This approach enabled the refinement of storage configurations to enhance temperature uniformity and energy efficiency. The fully packed storage configuration, designed based on standard industry practices, was modeled with the following specifications: the facility accommodates 80 pallets, each with two tiers, totaling 160 pallets (Figure 4). Each pallet holds 42 boxes arranged in a 6 × 7 tier configuration, resulting in a total of 6720 boxes (Figure 4b,c). Each cabbage box weighs between 13 and 15 kg, yielding an approximate total storage weight of 100 tons. The storage dimensions were set at 990 cm in width, 1710 cm in depth, and 850 cm in height, with the height optimized to enhance air circulation efficiency. Additionally, clearance gaps were maintained between pallets and walls to facilitate airflow and ease of movement.

**Figure 5:**
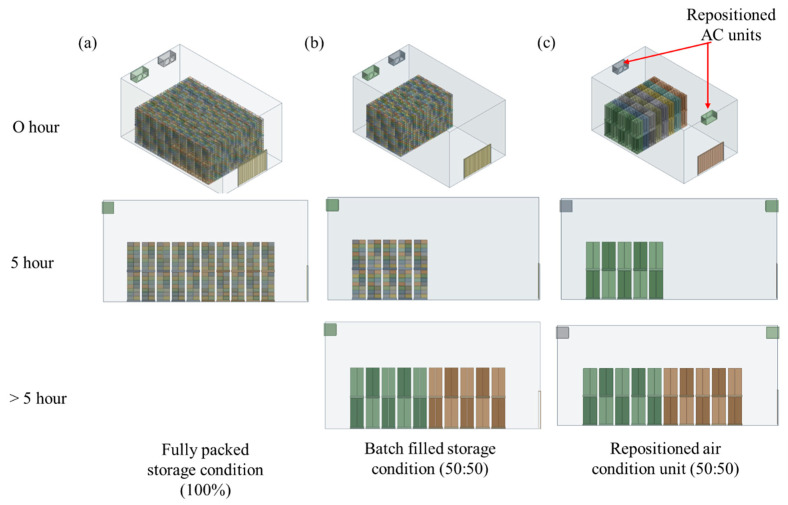
CFD simulation scenarios for optimizing cold storage conditions. (**a**) Standard condition fully packed, (**b**) standard condition with batch filling, and (**c**) repositioned air conditioning units with batch filling.

To ensure the applicability of the CFD model at a commercial scale, three operational scenarios were simulated. The first scenario, referred to as the “fully packed storage condition”, represented a storage modeled at full capacity (6720 boxes), providing baseline performance data (Figure 5a). The second scenario, “batch-filled storage condition”, simulated a partially filled loading process in which 50% of the cabbage boxes were initially loaded (i.e., the first set of baskets), with the remaining 50% added as the operation continued (second set of baskets) (Figure 5b). This scenario allowed for the analysis of how gradual changes in storage load influence air distribution and temperature over time. The third scenario, “batch filling with repositioned air conditioning units”, involved modifying the position of the air conditioning units to enhance cooling efficiency during the staged loading process (Figure 5c). These simulations were essential for scaling up the small-scale CFD model to a commercial storage setting. While small-scale models provide a controlled environment for studying heat and mass transfer, they do not fully capture the complex interactions of airflow and temperature gradients in large storage rooms. The CFD approach enabled a detailed assessment of these factors, allowing for the determination of optimal operating conditions for commercial CC storage.

By refining key parameters, such as airflow distribution, loading strategy, and cooling unit placement, this study provides a scientific framework for improving cold storage efficiency. The results demonstrate how computational modeling can guide practical improvements in commercial-scale storage facilities, ensuring uniform cooling, reducing energy costs, and extending the shelf life of stored cabbage.

## 3. Results and Discussion

### 3.1. K-Means Clustering Analysis of CC

**Figure 6:**
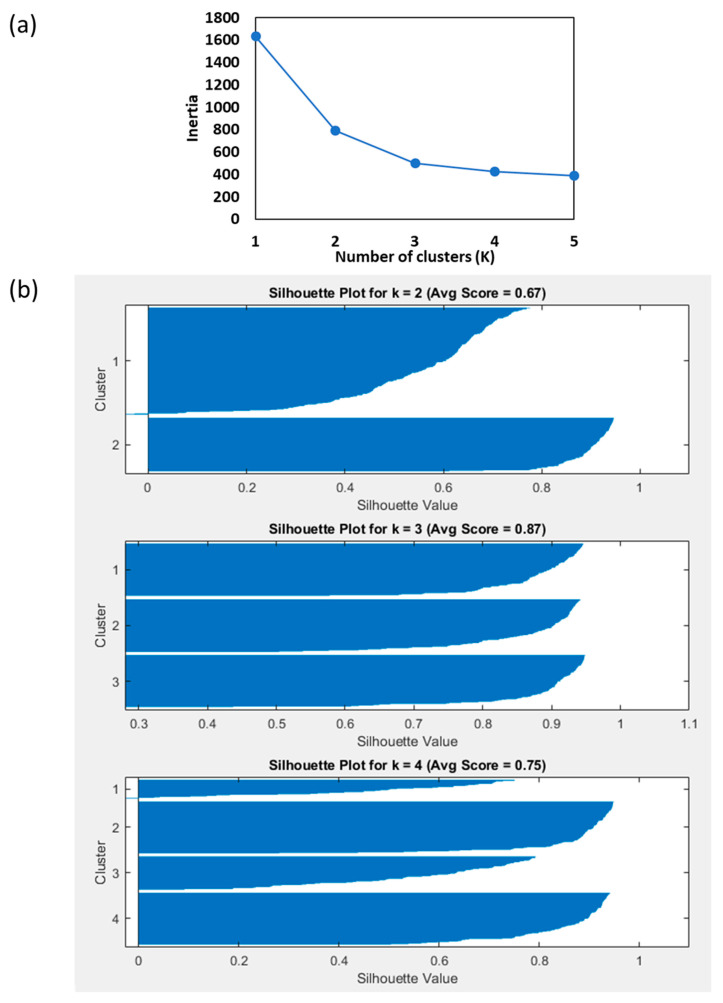
Determination of optimal K value and clustering validation for CC using silhouette analysis: (**a**) elbow method and (**b**) silhouette plot at K = 2, 3, and 4, respectively.

The K-means clustering analysis of CC morphological parameters is presented in Table 2. To determine the optimal number of clusters, the elbow method was applied, which revealed a clear inflection point at K = 3, suggesting three distinct groups based on the circumference length, circumference width, and weight. This was further validated using the silhouette method, which assesses the clustering quality by evaluating both the within-cluster cohesion and the between-cluster separation [29]. The silhouette scores for K = 2, 3, and 4 were 0.67, 0.87, and 0.75, respectively, with K = 3 achieving the highest score (Figure 6). The average silhouette coefficient across clusters for K = 3 confirmed its suitability as the optimal number of clusters.

Based on the results, class 1 showed the smallest dimensions followed by class 2 and 3. These findings demonstrate the ability of the K-means clustering algorithm to classify CC based on measurable morphological traits effectively. Similar clustering approaches have been applied to other vegetables, such as broccoli, where the elbow method identified three optimal clusters based on the crown diameter, stalk length, and weight, confirming the robustness of this technique across different produce types [30].

By segmenting CC into distinct morphological classes, the clustering results provide valuable insights for postharvest handling and packaging strategies. Tailoring the storage and transportation conditions based on class-specific properties could enhance the overall quality and shelf life of the produce. The combined use of the elbow and silhouette methods ensured a reliable framework for clustering analysis.

### 3.2. CO_2_, Relative Humidity, and Velocity in Storage Facility

**Figure 7:**
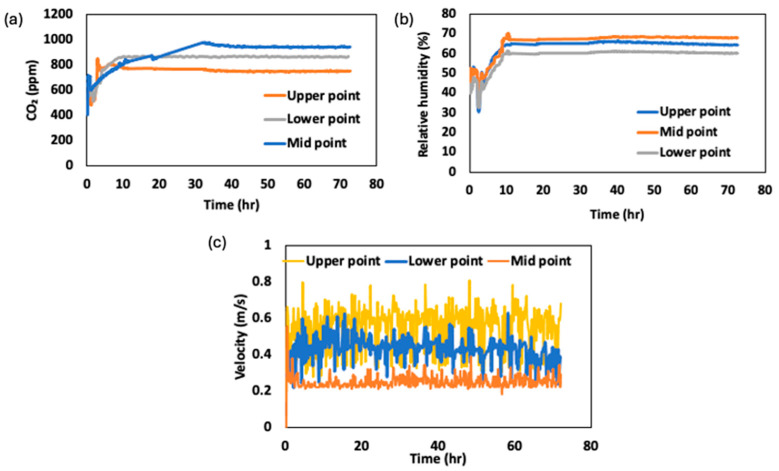
Changes in storage facility properties during a 72 h period: (**a**) CO_2_ concentration, (**b**) relative humidity, and (**c**) air velocity near the storage basket.

The results of the critical storage factors, such as CO_2_ concentration, relative humidity (RH), and air velocity at different positions within the storage facility, are shown in Figure 7. These measurements were taken at three distinct positions: lower (30 cm), mid (95 cm), and upper (120 cm) points located at the front of the stacked baskets. The result indicate that CO_2_ levels were highest at the mid-point layer of the storage facility, reaching an average concentration of 921 ± 8 ppm, which correlated with a reduced air velocity in localized environments (Figure 7c). These results align with findings from Mahajan et al. [31], who reported CO_2_ concentrations ranging from 800 to 1500 ppm in controlled atmosphere storage of fruits and vegetables, with higher levels observed in areas of restricted airflow. Similarly, Rao [32] noted that CO_2_ accumulation is strongly influenced by airflow patterns, with stagnant areas exhibiting higher CO_2_ concentrations, consistent with our observations.

The RH values remained relatively stable across different locations, averaging 59–68%, but were slightly higher in areas with limited airflow, such as the mid-point layer, where the RH reached 69.6%. This suggests that air circulation plays a critical role in moisture exchange and reduces water loss from transpiration. Comparable RH values were reported by Thompson et al. [33], who observed RH levels of 60–93% in cold storage facilities for leafy vegetables, with higher humidity in poorly ventilated zones. These findings emphasize the importance of a uniform airflow to maintain optimal RH levels and prevent moisture-related spoilage.

The measured air velocities within the storage facility exhibited significant variability, with average values of 0.45 ± 0.19 m/s at the lower position, 0.23 ± 0.11 m/s at the mid-point, and 0.53 ± 0.18 m/s at the upper position. This variability highlights the need for a uniform airflow to minimize respiration hotspots and ensure consistent storage conditions. Praeger et al. [34] reported similar air velocity ranges (0.1–0.4 m/s) between stack baskets of apples in cold storage facilities, noting that uneven air movement can lead to localized areas of higher respiration rates and moisture buildup, increasing the risk of spoilage. Our results further support this observation, as the mid-point layer, with the lowest air velocity (0.23 ± 0.11 m/s), also exhibited the highest CO_2_ concentration and RH, underscoring the interplay between these factors.

By integrating measurements of CO_2_, RH, and air velocity, this study underscores the complex interaction between storage environmental factors and the physiological processes of stored CC. The effective management of these parameters can enhance the energy efficiency in cold storage systems.

### 3.3. Effects of CC Heat of Respiration on the Heat Transfer Model in Storage Facility

**Figure 8:**
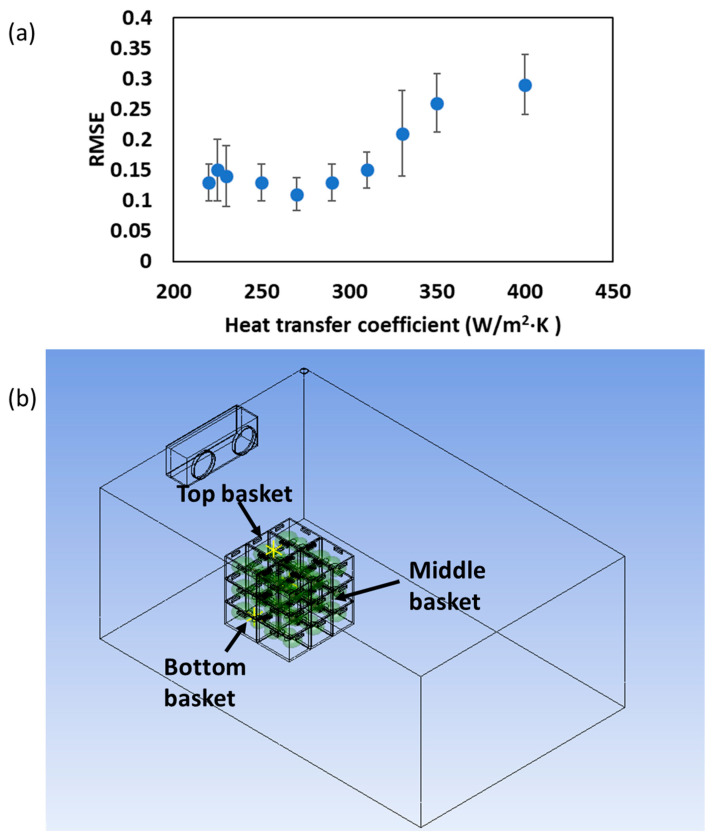
(**a**) Changes in average RMSE values based on heat transfer coefficient (h), and (**b**) schematic representation of the stacked CC basket arrangement inside a storage unit, indicating temperature measurement locations at the top, middle, and bottom baskets.

**Figure 9:**
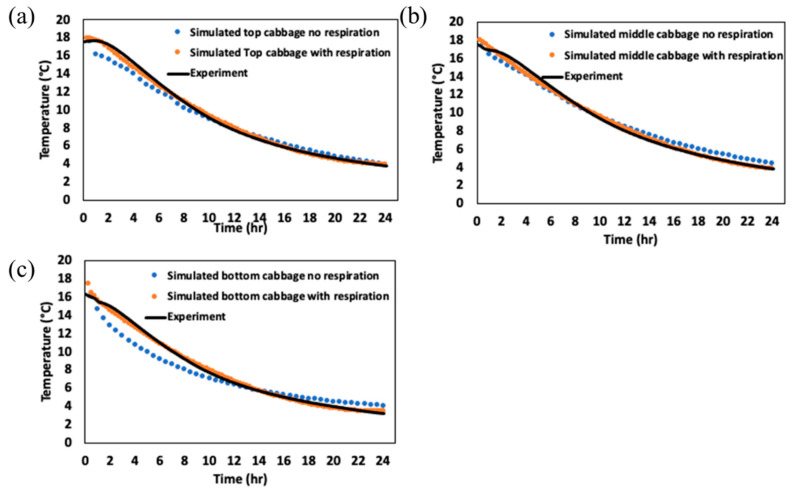
Comparison of heat transfer model with heat of respiration and without heat of respiration at different positions within the basket: (**a**) top basket, (**b**) middle basket, and (**c**) bottom basket.

The impact of the heat of respiration of CC on the accuracy of the heat transfer model in a small-scale storage facility is presented in this section. This study focused on determining an appropriate heat transfer coefficient (h) and validating the model by incorporating the heat of respiration to improve the temperature prediction accuracy (Figure 8 and Figure 9). To determine the optimal value for the heat transfer coefficient (h), simulations were performed with varying h values. The results revealed that the lowest root mean square error (RMSE) value occurred at 270 W/m^2^∙K (Figure 8a), demonstrating that this value of h yielded the most accurate temperature predictions. The temperature profiles at three different positions (i.e., top, middle, and lower CC basket) in the small-scale storage facility were simulated and compared with experimental results (Figure 8b).

The simulation model with the heat of respiration incorporated provided temperature predictions that closely matched the experimental data, with an RMSE value < 0.54 °C at all measured positions, confirming the significance of heat of respiration in the model (Figure 9). Similar studies have explored the role of respiration heat in modeling temperature fluctuations in storage environments. For example, Benitez et al. [35] studied the heat production of pineapple under various storage conditions (2~13 °C) over a 10-day period and found that respiration significantly influenced the internal temperature profiles, especially in the early stages of storage. They observed that models incorporating respiration heat led to more accurate predictions compared to those that ignored this factor. Similarly, Alexander et al. [8] demonstrated that including heat of respiration in simulation models improved the prediction of temperature changes in apples, particularly during the first few hours of storage, when respiration rates are highest. These studies support the findings from the current research, highlighting the importance of accounting for biological heat production in temperature modeling for stored produce.

The results from the current study also showed that the simulation model without respiration heat differed significantly from experimental values during the early storage period (0~10 h). This difference was likely due to the rapid increase in the respiration rate at the beginning of storage, which later stabilized as the material temperature cooled down, aligning the model with the experimental data as the storage period progressed. This study, therefore, demonstrates that incorporating the heat of respiration into heat transfer models improves the accuracy of temperature predictions in the storage facility for CC. The results confirmed that accounting for biological heat production is essential for more precise simulations, especially in the early stages of storage when respiration rates are highest.

### 3.4. Model Simplification for Storage Facility Scale-Up and Computational Efficiency

**Figure 10:**
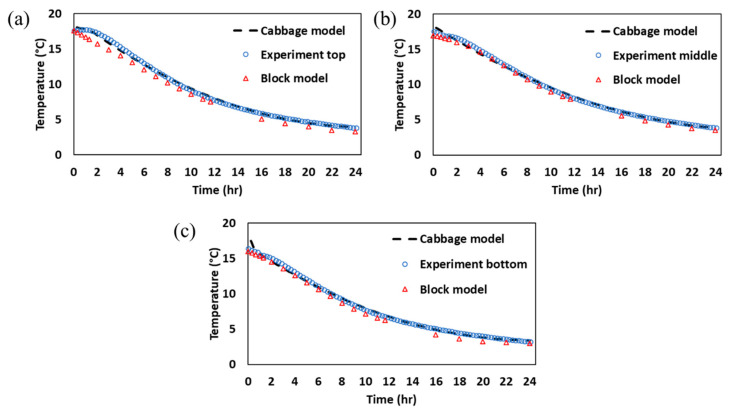
Comparison of heat transfer result in different geometry models at different positions within the basket: (**a**) top basket, (**b**) middle basket, and (**c**) bottom basket.

To facilitate computational efficiency in scaling up the small model for commercial-scale storage facilities, a systematic simplification approach was adopted. The small-scale model, which accurately predicted the temperature distribution using the actual cabbage geometry and incorporated the heat of respiration, was simplified into a block-based model, i.e., a model where the individual cabbages inside a basket were replaced with a single solid block representing the bulk properties of the cabbages. This simplification was critical to reduce computational costs while maintaining the accuracy of heat and mass transfer predictions. The block model was validated against experimental data and the detailed cabbage model to ensure its reliability for commercial-scale simulations (Figure 10). The results demonstrated that the block model closely matched the experimental data, with RMSE values of <0.89 °C for the top basket, <0.56 °C for the middle basket, and <0.61 °C for the bottom basket. These values were slightly higher than those of the detailed cabbage model (RMSE < 0.54 °C at all positions), but still within an acceptable range for practical applications. The slight increase in RMSE for the block model can be attributed to the simplification of the geometry, which may not fully capture the intricate airflow patterns and localized heat transfer effects around individual cabbages. However, the block model effectively replicated the overall thermal behavior, making it a suitable alternative for commercial-scale simulations.

The simplification of the model geometry significantly reduced the computational costs, as summarized in Table 3. The block model required approximately 38% less convergence time, 40% less overall simulation time, and 30% less RAM usage compared to the detailed cabbage model. The results demonstrated that the block model provides a balance between accuracy and computational efficiency, making it a practical tool for large-scale applications. This reduction in computational resources is critical for scaling up simulations to commercial storage facilities, where the complexity and size of the computational domain increase substantially. The block model’s efficiency enables faster iterations and more extensive parametric studies, which are essential for optimizing storage conditions in large-scale applications. Similar approaches to model simplification have been employed in other studies to enhance the computational efficiency. For example, Alexander et al. [8] simplified the geometry of apple stacks in cold storage simulations and found that block-based models could accurately predict temperature distributions while reducing the computational time by up to 30%. Similarly, Hoang et al. [36] demonstrated that simplified models of packaged produce in refrigerated containers provided reliable predictions of thermal behavior with significantly lower computational costs. These studies support the findings of the current research, highlighting the practicality of using simplified models for commercial-scale simulations without compromising accuracy.

### 3.5. Evaluating Storage Conditions for Optimal Design of Commercial-Scale CC Storage Room

**Figure 11:**
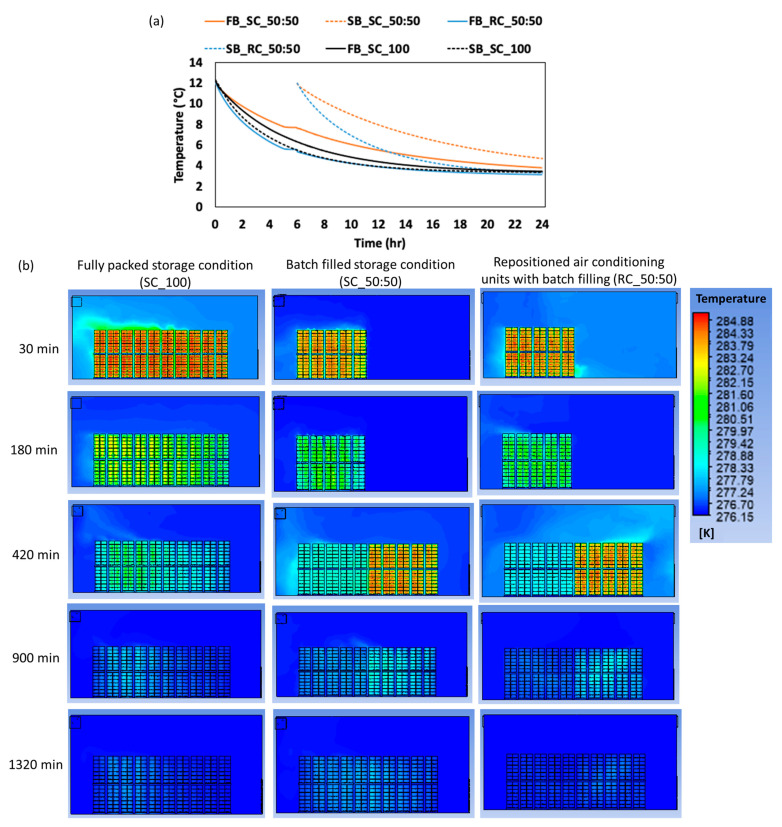
Temperature distribution in the CC storage facility under different operational conditions: (**a**) temperature profiles over time and (**b**) contour images showing the hot zone regions in the facility. [FB_SC_50:50 and SB_SC_50:50 represent the first and second sets of baskets under batch filling conditions, respectively; FB_RC_50:50 and SB_RC_50:50 represent the first and second sets of baskets under repositioned air conditioning with batch filling, respectively; and FB_SC_100 and SB_SC_100 represent the first and second sets of baskets under fully packed storage condition, respectively.

**Figure 12:**
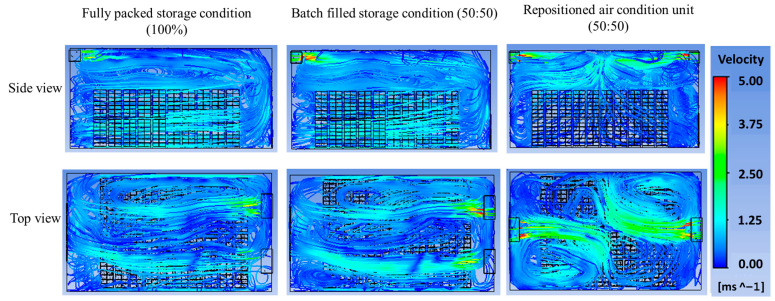
Airflow patterns in the CC storage facility under varying conditions.

To identify the most effective storage conditions for CC, three operational scenarios were analyzed: (a) fully packed storage, (b) batch-filled storage (50:50), and (c) repositioned air conditioning units with batch filling. These scenarios were evaluated based on the temperature distribution, airflow patterns, and cooling efficiency (Figure 11 and Figure 12). The goal was to determine the conditions that minimize temperature gradients, reduce hot zones, and achieve uniform cooling. The temperature gradient refers to the time required for CC baskets to cool to 4.25 °C, while the equilibrium time is the time taken to stabilize at the target storage temperature of 4.1 °C. Hot zones are areas with higher localized temperatures due to insufficient cooling.

This study addresses the challenges of airflow obstruction and uneven cooling caused by densely packed storage arrangements, which have often been overlooked in the existing literature. The storage facility model was developed using the validated CFD block model from the small-scale study (Section 3.4), incorporating key variables such as the heat of respiration, airflow velocities, and temperature effects as boundary conditions. This ensured an accurate reflection of real-world conditions, particularly during the early storage period when respiration rates are highest.

Batch filling was introduced to address the limitations of fully packed designs, where dense stacking obstructs the airflow and creates uneven cooling. In this strategy, the first set of CC baskets (3360 boxes) was placed in the storage facility for 5 h before introducing the second set. Figure 11a illustrates the temperature distribution at the center block under the three scenarios. In the fully packed condition, the largest hot zone is observed at the center of the first basket set, indicating poor air circulation due to dense packing (Figure 11b). In the batch-filled condition, the hot zone shifts to the center of the second basket set, showing an improved but still limited cooling efficiency. However, in the scenario with repositioned air conditioning units and batch filling, hot zones dissipated rapidly, achieving the most uniform temperature distribution (Figure 11b). This configuration minimized localized temperature spikes, which is critical for maintaining the produce quality and reducing spoilage risks.

The airflow analysis provided further insights into the differences between the storage conditions (Figure 12). In the fully packed design, the airflow collided with the walls opposite the air conditioning unit before reflecting toward the baskets, resulting in reduced air velocity and limited circulation. This inefficient airflow pattern is consistent with findings from Ferrua and Singh [37], who observed that densely packed produce stacks often lead to poor air circulation and localized hot spots. In contrast, the repositioned air conditioning units with batch filling directed the airflow more effectively between the CC baskets, minimizing stagnant areas and enhancing cooling performance. This configuration ensured that cold air reached all parts of the storage facility, reducing temperature gradients and improving the overall cooling efficiency. This aligns with Alexander et al. [8], who emphasized the importance of optimizing airflow patterns to achieve uniform cooling in storage facilities.

Table 4 provides a quantitative comparison of temperature gradient and equilibrium time across the three conditions. Under the fully packed condition, the first CC baskets required 12 h and 55 min to reach 4.25 °C, while the second baskets took 15 h and 30 min. The equilibrium times for these baskets were 21 h and 30 min and 18 h and 40 min, respectively. In contrast, the repositioned air conditioning units with batch filling significantly reduced both the temperature gradient and equilibrium time. For instance, the first baskets reached 4.25 °C in 11 h and 10 min and stabilized at 4.1 °C in 17 h and 15 min. These results align with findings from other studies; for example, Xin et al. [38] investigated the airflow and cooling efficiency of air conditioner installation at different positions in a large laboratory and demonstrated that repositioning the air conditioning units improved the airflow uniformity and reduced the cooling time. Similarly, Nalbandi et al. [39] presented an innovative parallel airflow system for force-air cooling of strawberries, similar to the design in the current study. The authors confirmed that the modified parallel airflow system improved the uniformity of the cooling process in strawberries, thus confirming the importance of optimizing the air conditioning placement and adopting batch-filling strategies to improve the cooling efficiency in CC storage facilities.

## 4. Conclusions

This study optimized Chinese cabbage (CC) storage design by integrating K-means clustering, heat transfer analysis, and respiratory heat effects. The morphological properties of CC showed significant variability, which were effectively classified into three clusters using K-means clustering, with a silhouette coefficient of 0.87. Key environmental parameters, including CO_2_ concentration, relative humidity (RH), and air velocity, were analyzed within the storage facility. CO_2_ levels were highest at the mid-point layer due to reduced airflow, while the RH remained stable but slightly elevated in poorly ventilated areas, emphasizing the need for uniform air circulation. Heat transfer modeling was conducted with and without respiration heat. Including respiration improved the model accuracy, with RMSE values < 0.54 °C. Excluding it led to significant temperature deviations during the early stage of storage (0–10 h) due to high initial respiration rates. A small-scale validation yielded a modified block geometry model for commercial-scale storage analysis, balancing the accuracy and computational efficiency, and reducing the convergence time by 38% and RAM usage by 30%. To optimize commercial storage, three designs were evaluated: fully packed, batch-filled (50:50), and repositioned air conditioning with batch filling. The temperature gradients were significantly reduced under batch filling, with an equilibrium (4.1 °C) reached faster in the repositioned air conditioning design (17 h 15 min) compared to the fully packed storage (21 h 30 min). The repositioned design also minimized hot zones and improved airflow distribution. This study underscores the importance of integrating CC’s morphological variability, environmental factors, and respiratory heat into storage design to enhance the cooling efficiency and product quality, providing a practical framework for commercial vegetable storage optimization.

## Figures and Tables

**Table 1:** The thermal and physical properties of CC.

Properties	Class 1	Class 2	Class 3
Thermal conductivity (k) (W/m·K)	8.1 × 10^−5^ T + 2.2 × 10^−2^	8.3 × 10^−5^ T + 2.27 × 10^−2^	8.5 × 10^−5^ T + 2.3 × 10^−2^
Specific heat (C_P_) (J/kg·K)	−0.0048 T^2^ + 1.76 T + 1770	−0.00475 T^2^ + 1.765 T + 1775	−0.0047 T^2^ + 1.78 T + 1780
Density (ρ) (kg/m^3^)	601.64 ± 42.68	675.70 ± 38.59	754.34 ± 35.34

**Table 2:** Cabbage morphological parameter values for each class.

Properties		Class 1	Class 2	Class 3
	Max	79	84	93
Length circumference (cm)	Min	68	80	85
	Average	73.32 ± 3.34	82.67 ± 1.17	89.17 ± 2.45
	Max	48	54	61
Width circumference (cm)	Min	44	49	55
	Average	46.73 ± 2.24	51.89 ± 2.37	58.67 ± 2.77
	Max	1783.26	2483.57	3112.85
Weight (g)	Min	1306.39	1795.23	2484.77
	Average	1503.20 ± 118.39	2132.48 ± 127.16	2826.37 ± 121.25

**Table 3:** Computational cost reduction achieved by model simplification.

Properties	Cabbage Model	Block Model	Reduction (%)
Number of mesh element	2,694,876	2,102,003	22
Convergence time (h)	2.1	1.3	38
Simulation time (h)	4.5	2.7	40
RAM uasge (GB)	32	22.4	30

**Table 4:** Comparison of temperature gradient and equilibrium time across different storage conditions for CC.

Loading Process	Model	Temperature Gradient		Equilibrium Temperature	
		Time	Temp (°C)	Time	Temp (°C)
Fully packed storage condition	First block	12 h 55 min	4.25 °C	21 h 30 min	4.1 °C
	Second block	15 h 30 min	4.25 °C		
Batch-filled storage condition (50:50)	First block	12 h 05 min	4.25 °C	18 h 40 min	4.1 °C
	Second block	14 h 35 min	4.25 °C		
Repositioned air condition unit with batch filling (50:50)	First block	11 h 10 min	4.25 °C	17 h 15 min	4.1 °C
	Second block	11 h 10 min	4.25 °C		

## Data Availability

The data presented in this study are available on request from the corresponding author.

## References

[1] Dahiru S., Salele Y., Japhet A.T., Abubakar M.Y., Adam A.B. (2024). Nutritional status and risk characterization of red pepper, cabbage, lettuce and spinach grown at Ajiwa, Batagarawa, Lambun Sarki and Kofar Marusa vegetable farms, Katsina State, Nigeria. Earthline J. Chem. Sci..

[2] Zhang R., He Q., Pan Q., Feng Y., Shi Y., Li G., Khan A. (2024). Blue-green light treatment enhances the quality and nutritional value in postharvest Chinese cabbage (*Brassica rapa* L. ssp. *pekinensis*). Food Chem. X.

[3] Ta-Oun P., Suwakrai K., Hongpakdee P., Nimkingrat P. (2021). The impact of bio-control management, storage temperature and packaging on postharvest characterization of Chinese flowering cabbage. Acta Hortic..

[4] Ma Y., Zhang W., Cheng S., Liu Y., Yang W., Wang Y., Chen G. (2022). Postharvest storage at near-freezing temperature maintained the quality and antioxidant properties of *Prunus domestica* L. cv. Ximei fruit. Sci. Hortic..

[5] Lim K.T., Kim J., Chung J.H. (2014). Development of Long-Term Storage Technology for Chinese Cabbage—Physiological Characteristics of Postharvest Freshness in a Cooler with a Monitoring and Control Interface. J. Biosyst. Eng..

[6] Oyinloye T.M., Yoon W.B. (2024). Analysis of Mass Transfer and Shrinkage Characteristics of Chinese Cabbage (*Brassica rapa* L. ssp. *pekinensis*) Leaves during Osmotic Dehydration. Foods.

[7] Chaves P.J.L., Cepeda J.T., Álvarez F.J.G., Orzáez M.J.H. (2020). Influence of moisture, temperature and microbial activity in biomass sustainable storage. Special Focus on Olive Biomasses. Int. J. Environ. Sci. Nat. Res..

[8] Alexander L.D., Jakhar S., Dasgupta M.S. (2024). Optimizing cold storage for uniform airflow and temperature distribution in apple preservation using CFD simulation. Sci. Rep..

[9] Getahun S.T. (2017). Investigating Cooling Performance and Energy Utilization of Refrigerated Shipping Container Packed with Fresh Fruit Using Computational Fluid Dynamics Modelling. Ph.D. Thesis.

[10] Arpaci E., Atayılmaz Ş.Ö., Gemici Z. (2024). Exploring Mathematical Modeling and CFD in Convective Drying of Fruits and Vegetables: A Review. Food Bioprocess Technol..

[11] Yin J., Guo M., Liu G., Ma Y., Chen S., Jia L., Liu M. (2022). Research progress in simultaneous heat and mass transfer of fruits and vegetables during precooling. Food Eng. Rev..

[12] Prukwarun W., Khumchoo W., Seancotr W., Phupaichitkun S. (2013). CFD simulation of fixed bed dryer by using porous media concepts: Unpeeled longan case. Int. J. Agric. Biol. Eng..

[13] Halder A. (2010). A Framework for Multiphase Heat and Mass Transport in Porous Media with Applications to Food Processes. Ph.D. Thesis.

[14] Sajadiye S.M., Ahmadi H., Zolfaghari M., Mohtasebi S.S., Mostofi Y., Raja A. (2013). A multi-scale three-dimensional CFD model of a full loaded cool storage. Int. J. Food Eng..

[15] Worasawate D., Sakunasinha P., Chiangga S. (2022). Automatic classification of the ripeness stage of mango fruit using a machine learning approach. AgriEngineering.

[16] Liming X., Yanchao Z. (2010). Automated strawberry grading system based on image processing. Comput. Electron. Agric..

[17] Kolodziejczyk M., Butrymowicz D., Smierciew K., Gagan J. Numerical Modelling of Heat and Mass Transfer Processes in Chinese Cabbage Cold Storage Chamber. Proceedings of the International Refrigeration and Air Conditioning Conference.

[18] Priss O., Yevlash V., Zhukova V., Kiurchev S., Verkholantseva V., Kalugina I., Bandurenko H. (2017). Investigation of the respiration rate during storage of fruit vegetables under the influence of abiotic factors. EUREKA Life Sci..

[19] González-Buesa J., Salvador M.L. (2019). An Arduino-based low cost device for the measurement of the respiration rates of fruits and vegetables. Comput. Electron. Agric..

[20] Kandasamy P. (2022). Respiration rate of fruits and vegetables for modified atmosphere packaging: A mathematical approach. J. Postharvest Technol..

[21] Al-Ati T., Hotchkiss J.H. (2002). Application of packaging and modified atmosphere to fresh-cut fruits and vegetables. Fresh-Cut Fruits and Vegetables: Science, Technology, and Market.

[22] ASHRAE (2006). Thermal properties of foods, In Handbook—Refrigeration.

[23] ANSYS Inc (2009). ANSYS FLUENT 12.0 User’s Guide. Section 7.2.2 Solid Conditions. https://www.afs.enea.it/project/neptunius/docs/fluent/html/ug/node232.htm.

[24] Bazgaou A., Fatnassi H., Bouharroud R., Tiskatine R., Wifaya A., Demrati H., Bouirden L. (2023). CFD modeling of the microclimate in a greenhouse using a rock bed thermal storage heating system. Horticulturae.

[25] Oyinloye T.M., An S., Cho C.W., Yoon W.B. (2024). Optimization of Steaming Conditions for Bellflower Root (*Platycodon grandiflorus*) Using K-Means Clustering-Based Morphological Grading System. Processes.

[26] Zhao Y., Xie J. (2004). Practical applications of vacuum impregnation in fruit and vegetable processing. Trends Food Sci. Tech..

[27] Park S.H., Seo D.H., Jeong J.H. (2020). Experimental and numerical analysis of thermal flow in open-cell porous metal during Darcy-Forchheimer transition regime. Appl. Therm. Eng..

[28] Kosari E., Vafai K. (2021). Synthesis of flow and thermal transport in porous media as applied to biological applications. J. Heat Transf..

[29] Rousseeuw P.J. (1987). Silhouettes: A graphical aid to the interpretation and validation of cluster analysis. J. Comput. Appl. Math..

[30] Merliana N.P.E., Santoso A.J. Analisa Penentuan Jumlah Cluster Terbaik pada Metode K-Means Clustering. Proceedings of the National Seminar on Multidisciplinary Science.

[31] Mahajan P.V., Caleb O.J., Singh Z., Watkins C.B., Geyer M. (2014). Postharvest treatments of fresh produce. Philos. Trans. R. Soc. A Math. Phys. Eng. Sci..

[32] Rao C.G. (2015). Engineering for Storage of Fruits and Vegetables: Cold Storage, Controlled Atmosphere Storage, Modified Atmosphere Storage.

[33] Thompson A.K., Prange R.K., Bancroft R., Puttongsiri T. (2018). Controlled Atmosphere Storage of Fruit and Vegetables.

[34] Praeger U., Jedermann R., Sellwig M., Neuwald D.A., Hartgenbusch N., Borysov M., Geyer M. (2020). Airflow distribution in an apple storage room. J. Food Eng..

[35] Benítez S., Chiumenti M., Sepulcre F., Achaerandio I., Pujolá M. (2012). Modeling the effect of storage temperature on the respiration rate and texture of fresh cut pineapple. J. Food Eng..

[36] Hoang M.L., Verboven P., Baelmans M., Nicolaï B.M. (2003). A continuum model for airflow, heat and mass transfer in bulk of chicory roots. Trans. ASAE.

[37] Ferrua M.J., Singh R.P. (2009). Modeling the forced-air cooling process of fresh strawberry packages, Part II: Experimental validation of the flow model. Int. J. Refrig..

[38] Xin S., Xu H., Li S., Wang W., Guo J., Yang W. (2020). Efficiency evaluation of a floor standing air conditioner with different installation positions and air supply parameters applied to a large laboratory. J. Build. Eng..

[39] Nalbandi H., Seiiedlou S., Ghasemzadeh H.R., Rangbar F. (2016). Innovative parallel airflow system for forced-air cooling of strawberries. Food Bioprod. Process..

